# Supplementary material for: Evaluation of the Second Premolar’s Bud Position Using Computer Image Analysis and Neural Modelling Methods
Source: Int J Environ Res Public Health. 2022 Nov 18;19(22):15240. doi: 10.3390/ijerph192215240 (PMC9691188; doi:10.3390/ijerph192215240)
Supplement: Supplementary file 1 [file ijerph-19-15240-s001.zip › ijerph-2027437-supplementary.pdf]

**Table S1.** Matrix of learning set variables LS 2021,04,25 Q I-IV.

| LS 2021,04,25 Q- I | LS 2021,04,25 Q- II | LS 2021,04,25 Q- III | LS 2021,04,25 Q- IV |
|---|---|---|---|
| GENDER | GENDER | GENDER | GENDER |
| AGE | AGE | AGE | AGE |
| AV | AV | AV | AV |
| CQ | CQ | CQ | CQ |
| CS | CS | CS | CS |
| CU | CU | CU | CU |
| CW | CW | CW | CW |
| CY | CY | CY | CY |
| DA | DA | DA | DA |
| DC | DC | DC | DC |
| DE | DE | DE | DE |
| DG | DK | DS | DO |
| DW | DY | EC | EA |
| ED | EE | EG | EF |
| EL | EP | EX | ET |
| DI | DM | DU | DQ |

**Table S2.** The effect of the neural modeling performed for the set LS 2021,04,25 Q- I.

| Nr. | Type | Quality lear. | Quality valid. | Quality test. | Error lear. | Error valid. | Error test. | Learning |
|---|---|---|---|---|---|---|---|---|
| **1** | MLP 1:1-1-1:1 | 0,9357 | 0,9247 | 0,9310 | 0,1678 | 0,1405 | 0,2159 | BP100, CG20, CG1b |
| **2** | MLP 3:3-8-3-1:1 | 0,8952 | 0,8199 | 0,7891 | 0,1604 | 0,1202 | 0,1833 | BP100, CG20, CG4b |
| **3** | MLP 5:5-10-4-1:1 | 0,7947 | 0,7779 | 0,7011 | 0,1423 | 0,1139 | 0,1634 | BP100, CG20, CG33b |
| **4** | MLP 9:9-8-1:1 | 0,7453 | 0,7525 | 0,7133 | 0,1334 | 0,1102 | 0,1656 | BP100, CG20, CG48b |
| **5** | MLP 8:8-5-1:1 | 0,7987 | 0,7376 | 0,7134 | 0,1432 | 0,1085 | 0,1654 | BP100, CG20, CG3b |
| **6** | RBF 14:14-5-1:1 | 0,9116 | 0,8711 | 0,8923 | 0,0958 | 0,0770 | 0,1210 | KM, KN, PI |
| **7** | RBF 14:14-15-1:1 | 0,8066 | 0,8371 | 0,7453 | 0,0848 | 0,0724 | 0,1017 | KM, KN, PI |
| **8** | RBF 14:14-10-1:1 | 0,8263 | 0,8205 | 0,7695 | 0,0868 | 0,0715 | 0,1053 | KM, KN, PI |
| **9** | RBF 14:14-11-1:1 | 0,8370 | 0,8057 | 0,7796 | 0,0880 | 0,0701 | 0,1061 | KM, KN, PI |
| **10** | RBF 14:14-13-1:1 | 0,8333 | 0,7997 | 0,7843 | 0,0876 | 0,0690 | 0,1066 | KM, KN, PI |

**Table S3.** Sensitivity analysis of RBF 14:14-5-1:1 networks.

| | Rank | Quotient |
|---|---|---|
| DW | 1 | 1,025810 |
| GENDER | 2 | 1,022931 |
| CY | 3 | 1,019064 |
| AGE | 4 | 1,014891 |
| CS | 5 | 1,008216 |
| CQ | 6 | 1,002889 |
| DE | 7 | 1,002556 |
| DC | 8 | 1,002496 |
| CW | 9 | 1,000556 |
| CU | 10 | 0,99766 |
| AV | 11 | 0,99732 |
| DG | 12 | 0,99143 |
| ED | 13 | 0,98984 |
| EL | 14 | 0,98867 |

**Figure S1.** Diagram of the RBF 14:14-5-1:1 network defining the DI parameter.

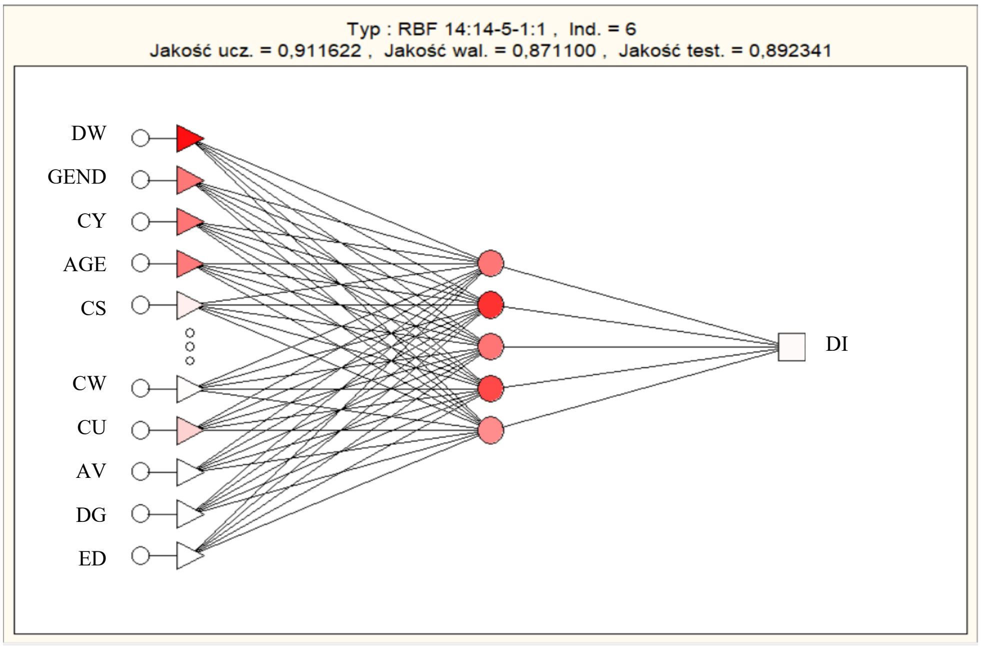

**Table S4.** The effect of the neural modeling performed for the LS 2021,04,25 Q- II set.

| Nr. | Type | Quality lear. | Quality valid. | Quality test. | Error lear. | Error valid. | Error test. | Learning |
|---|---|---|---|---|---|---|---|---|
| **1** | MLP 1:1-6-5-1:1 | 0,9732 | 0,9990 | 0,9731 | 0,2059 | 0,2162 | 0,3036 | BP100, CG20, CG0b |
| **2** | MLP 6:6-4-1:1 | 0,7482 | 0,8324 | 0,6971 | 0,1583 | 0,1793 | 0,2167 | BP100, CG20, CG4b |
| **3** | MLP 7:7-12-5-1:1 | 0,7287 | 0,8216 | 0,6716 | 0,1542 | 0,1783 | 0,2069 | BP100, CG20, CG6b |
| **4** | MLP 2:2-8-3-1:1 | 0,7782 | 0,8065 | 0,6659 | 0,1646 | 0,1768 | 0,2051 | BP100, CG20, CG2b |
| **5** | MLP 2:2-1-1:1 | 0,8476 | 0,8228 | 0,7659 | 0,1795 | 0,1756 | 0,2360 | BP100, CG20, CG14b |
| **6** | RBF 12:12-5-1:1 | 0,8808 | 0,9107 | 0,8888 | 0,0978 | 0,1028 | 0,1434 | KM, KN, PI |
| **7** | RBF 12:12-13-1:1 | 0,7602 | 0,8847 | 0,7145 | 0,0844 | 0,1017 | 0,1163 | KM, KN, PI |
| **8** | RBF 12:12-7-1:1 | 0,8496 | 0,8788 | 0,8317 | 0,0943 | 0,1001 | 0,1345 | KM, KN, PI |
| **9** | RBF 12:12-20-1:1 | 0,7288 | 0,8764 | 0,8339 | 0,0846 | 0,0980 | 0,1331 | KM, KN, PI |
| **10** | RBF 12:12-10-1:1 | 0,7367 | 0,8221 | 0,8039 | 0,0818 | 0,0940 | 0,1290 | KM, KN, PI |

**Table S5.** Sensitivity analysis of the RBF 12:12-5-1:1 network.

| | Rank | Quotient |
|---|---|---|
| AV | 1 | 1,026139 |
| DK | 2 | 1,020221 |
| DY | 3 | 1,019059 |
| AGE | 4 | 1,016806 |
| DE | 5 | 0,999930 |
| CS | 6 | 0,999540 |
| DC | 7 | 0,998969 |
| EE | 8 | 0,997174 |
| EP | 9 | 0,995340 |
| CW | 10 | 0,98631 |
| CU | 11 | 0,98397 |
| GENDER | 12 | 0,96540 |

**Figure S2.** Diagram of the RBF 12:12-5-1:1 network defining the DM parameter

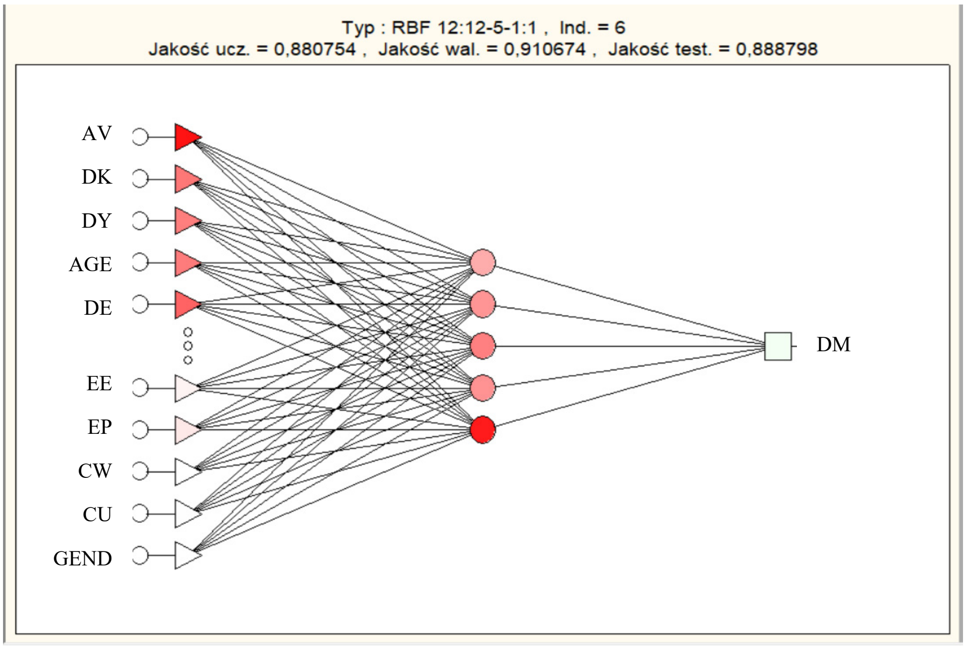


**Table S6.** The effect of the neural modeling performed for the LS 2021,04,25 Q- III set.

| Nr. | Type | Quality lear. | Quality valid. | Quality test. | Error lear. | Error valid. | Error test. | Learning |
|---|---|---|---|---|---|---|---|---|
| **1** | MLP 1:1-5-1:1 | 0,8433 | 0,8769 | 0,8591 | 0,1241 | 0,1239 | 0,1362 | BP100, CG20, CG3b |
| **2** | MLP 1:1-8-4-1:1 | 0,6930 | 0,8040 | 0,7216 | 0,1019 | 0,1137 | 0,1135 | BP100, CG20, CG11b |
| **3** | MLP 4:4-4-1:1 | 0,5351 | 0,6316 | 0,5738 | 0,0788 | 0,0892 | 0,0902 | BP100, CG20, CG38b |
| **4** | MLP 9:9-7-1:1 | 0,4297 | 0,5236 | 0,4618 | 0,0633 | 0,0740 | 0,0728 | BP100, CG20, CG63b |
| **5** | MLP 11:11-13-10-1:1 | 0,4533 | 0,5190 | 0,5042 | 0,0667 | 0,0733 | 0,0801 | BP100, CG20, CG55b |
| **6** | RBF 13:13-10-1:1 | 0,6777 | 0,7428 | 0,6890 | 0,0657 | 0,0692 | 0,0722 | KM, KN ,PI |
| **7** | RBF 13:13-20-1:1 | 0,6125 | 0,6459 | 0,6643 | 0,0603 | 0,0615 | 0,0720 | KM, KN ,PI |
| **8** | RBF 13:13-36-1:1 | 0,4563 | 0,6469 | 0,5637 | 0,0443 | 0,0603 | 0,0590 | KM, KN ,PI |
| **9** | RBF 13:13-47-1:1 | 0,4434 | 0,6279 | 0,5306 | 0,0430 | 0,0585 | 0,0567 | KM, KN ,PI |
| **10** | RBF 13:13-31-1:1 | 0,5260 | 0,6119 | 0,5954 | 0,0510 | 0,0571 | 0,0617 | KM, KN ,PI |

**Table S7.** Sensitivity analysis of RBF 13:13-10-1:1 networks.

| | Rank | Quotient |
|---|---|---|
| EG | 1 | 1,064450 |
| GENDER | 2 | 1,058868 |
| AV | 3 | 1,027203 |
| EC | 4 | 1,016206 |
| CW | 5 | 1,013149 |
| DS | 6 | 1,012426 |
| AGE | 7 | 1,011831 |
| DA | 8 | 1,008238 |
| CY | 9 | 1,005744 |
| DE | 10 | 1,00292 |
| DC | 11 | 0,99968 |
| CS | 12 | 0,99941 |
| EX | 13 | 0,97755 |

**Figure S3.** Diagram of the RBF 12:12-5-1:1 network defining the DU parameter.

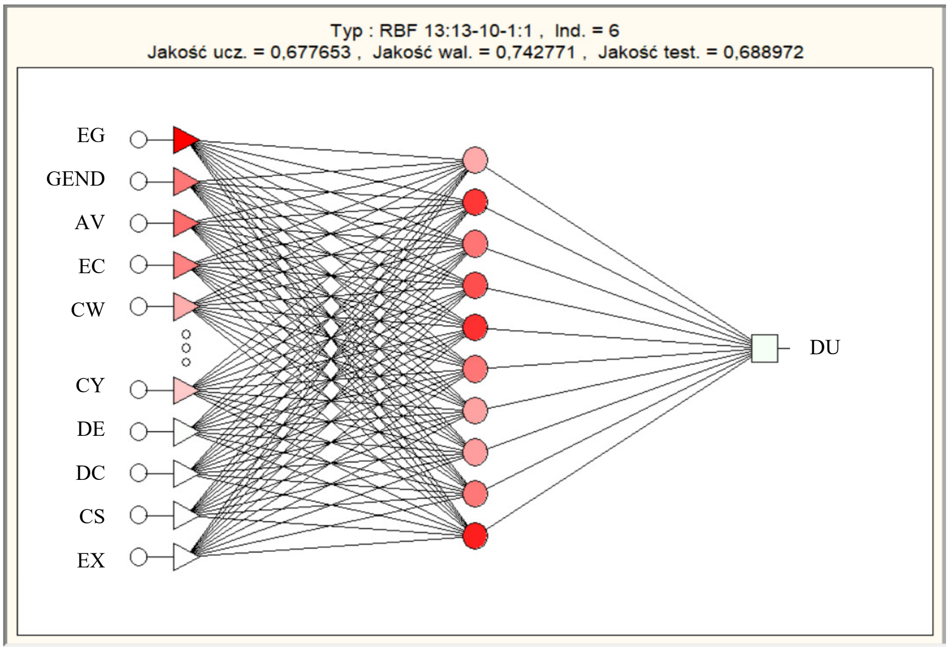

**Table S8.** The effect of the neural modeling performed for the LS 2021,04,25 Q-III set.

| Nr. | Type | Quality lear. | Quality valid. | Quality test. | Error lear. | Error valid. | Error test. | Learning |
|---|---|---|---|---|---|---|---|---|
| **1** | MLP 1:1-8-1:1 | 0,8660 | 0,8434 | 0,8249 | 0,1647 | 0,1896 | 0,1608 | BP100c,CG20, CG24b |
| **2** | MLP 1:1-7-2-1:1 | 0,6846 | 0,7318 | 0,6562 | 0,1302 | 0,1664 | 0,1284 | BP100, CG20, CG21b |
| **3** | MLP 4:4-4-1:1 | 0,5106 | 0,6237 | 0,6110 | 0,0971 | 0,1403 | 0,1197 | BP100, CG20, CG107b |
| **4** | MLP 11:11-7-1:1 | 0,5212 | 0,5437 | 0,5703 | 0,0992 | 0,1237 | 0,1126 | BP100, CG20, CG49b |
| **5** | MLP 6:6-4-1:1 | 0,4835 | 0,4788 | 0,4668 | 0,0920 | 0,1087 | 0,0935 | BP100,CG20,CG132b |
| **6** | RBF 10:10-10-1:1 | 0,6280 | 0,7221 | 0,7255 | 0,0655 | 0,0899 | 0,0776 | KM, KN, PI |
| **7** | RBF 10:10-20-1:1 | 0,5780 | 0,6788 | 0,6665 | 0,0606 | 0,0849 | 0,0731 | KM, KN, PI |
| **8** | RBF 10:10-23-1:1 | 0,5851 | 0,6732 | 0,6625 | 0,0610 | 0,0848 | 0,0714 | KM, KN, PI |
| **9** | RBF 10:10-25-1:1 | 0,5180 | 0,6267 | 0,6353 | 0,0540 | 0,0772 | 0,0685 | KM, KN, PI |
| **10** | RBF 10:10-27-1:1 | 0,5188 | 0,6147 | 0,6651 | 0,0541 | 0,0762 | 0,0724 | KM, KN, PI |

**Table S9.** Sensitivity analysis of RBF network 10:10-10-1:1.

| | Rank | Quotient |
|---|---|---|
| EA | 1 | 1,102474 |
| AV | 2 | 1,041425 |
| AGE | 3 | 1,027120 |
| EF | 4 | 1,017095 |
| ET | 5 | 1,017007 |
| DO | 6 | 1,012029 |
| CS | 7 | 1,003954 |
| CQ | 8 | 0,998992 |
| CW | 9 | 0,996286 |
| GENDER | 10 | 0,98011 |

**Figure S4.** Diagram of the RBF 10:10-10-1:1 network defining the DQ parameter.

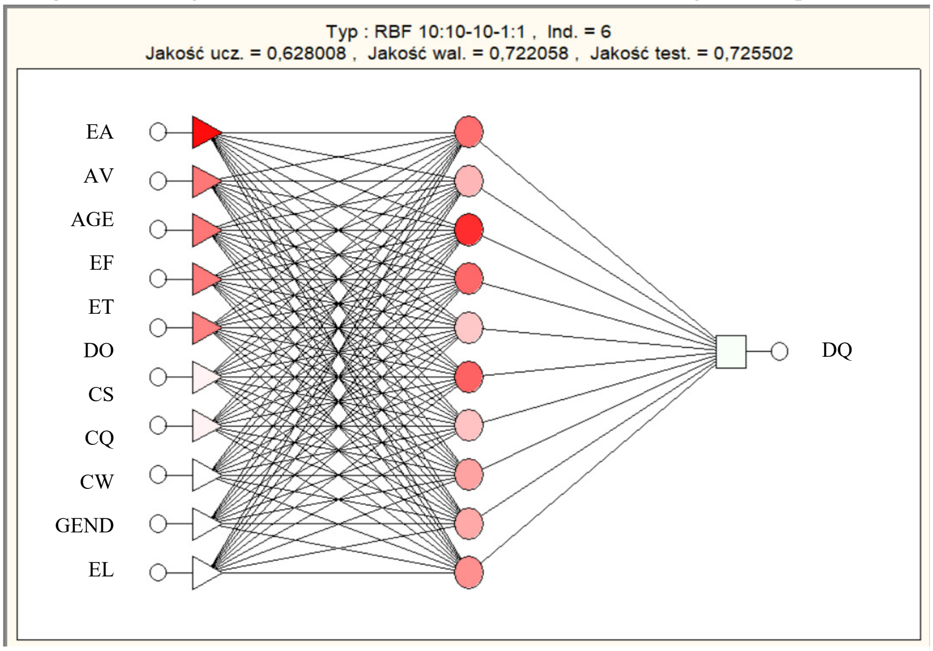


**Table S10.** Summary of generated models and their quality characteristics.

| Quadrant | I | II | III | IV |
|---|---|---|---|---|
| The model | RBF 14:14-5-1:1 | RBF 12:12-5-1:1 | RBF 13:13-10-1:1 | RBF 10:10-10-1:1 |
| Quality for the learning set | 0,8923 | 0,8888 | 0,689 | 0,7255 |
| Error *RMSE* for the learning set | 0,121 | 0,1434 | 0,0722 | 0,0776 |

**Table S11.** Summary of the variables used in the neural modeling process for each quadrant, the variables necessary for the operation of the model are marked in gray.

| **Quadrant** | I | II | III | IV |
|---|---|---|---|---|
| Model | RBF 14:14-5-1:1 | RBF 12:12-5-1:1 | RBF 13:13-10-1:1 | RBF 10:10-10-1:1 |
| Variable | | | | |
| 1 | AGE | AGE | AGE | AGE |
| 2 | GENDER | GENDER | GENDER | GENDER |
| 3 | AV | AV | AV | AV |
| 4 | CQ | CQ | CQ | CQ |
| 5 | CS | CS | CS | CS |
| 6 | CU | CU | CU | CU |
| 7 | CW | CW | CW | CW |
| 8 | CY | CY | CY | CY |
| 9 | DA | DA | DA | DA |
| 10 | DC | DC | DC | DC |
| 11 | DE | DE | DE | DE |
| 12 | DG | DK | DS | DO |
| 13 | DW | DY | EC | EA |
| 14 | ED | EE | EG | EF |
| 15 | EL | EP | EX | ET |
| Network output | DI | DM | DU | DQ |

**Table S12.** The effect of the neural modeling performed for the set of LS 2021,0425 4Q.

| Nr. | Type | Quality lear. | Quality valid. | Quality test. | Error lear. | Error valid. | Error test. | Learning |
|---|---|---|---|---|---|---|---|---|
| **1** | MLP 1:1-11-4:4 | 0,9803 | 0,9958 | 1,0555 | 0,1738 | 0,1666 | 0,1913 | BP100, CG20, CG31b |
| **2** | MLP 1:1-14-12-4:4 | 0,7629 | 0,9137 | 0,7082 | 0,1308 | 0,1448 | 0,1415 | BP100, CG20, CG95b |
| **3** | MLP 9:9-17-11-4:4 | 0,7556 | 0,7126 | 0,8172 | 0,1228 | 0,1266 | 0,1339 | BP100, CG20, CG23b |
| **4** | MLP 10:10-17-11-4:4 | 0,7372 | 0,7592 | 0,8136 | 0,1248 | 0,1264 | 0,1395 | BP100, CG20, CG47b |
| **5** | MLP 26:26-18-18-4:4 | 0,6233 | 0,7735 | 0,9944 | 0,0980 | 0,1261 | 0,2595 | BP100, CG20, CG10b |
| **6** | RBF 19:19-15-4:4 | 0,6895 | 0,8066 | 0,9140 | 0,0612 | 0,0728 | 0,0818 | KM, KN, PI |
| **7** | RBF 19:19-5-4:4 | 0,7542 | 0,8117 | 0,8593 | 0,0708 | 0,0728 | 0,0842 | KM, KN, PI |
| **8** | RBF 19:19-10-4:4 | 0,7137 | 0,7967 | 0,8467 | 0,0654 | 0,0719 | 0,0801 | KM, KN, PI |
| **9** | RBF 19:19-13-4:4 | 0,7149 | 0,7950 | 0,8436 | 0,0639 | 0,0714 | 0,0762 | KM, KN, PI |
| **10** | RBF 19:19-11-4:4 | 0,7107 | 0,7844 | 0,8491 | 0,0629 | 0,0707 | 0,0797 | KM, KN, PI |

**Table S13.** Sensitivity analysis of the RBF 19:19-5-4:4 network.

| | Ranga | Iloraz |
|---|---|---|
| DS | 1 | 1,018318 |
| EC | 2 | 1,016550 |
| DW | 3 | 1,013011 |
| EA | 4 | 1,012940 |
| DY | 5 | 1,011573 |
| AV | 6 | 1,010975 |
| DO | 7 | 1,007101 |
| AGE | 8 | 1,004293 |
| DK | 9 | 1,003046 |
| EE | 10 | 1,00232 |
| GENDER | 11 | 1,00219 |
| EF | 12 | 1,00186 |
| EX | 13 | 0,99895 |
| ED | 14 | 0,99832 |
| EG | 15 | 0,99739 |
| CQ | 16 | 0,99660 |
| DC | 17 | 0,99578 |
| EP | 18 | 0,99568 |
| ET | 19 | 0,98950 |

**Figure S5.** Diagram of the RBF 19:19-5-4:4 network specifying the parameter.

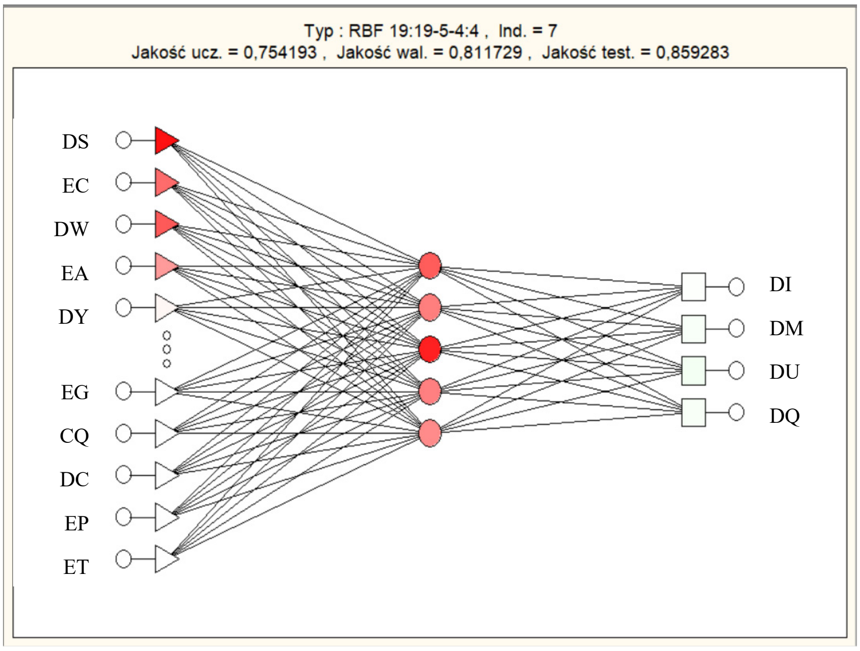

**Table S14.** Summary of generated ANN models. Networks ranked by quality for the learning set (descending) and by RMSE error for the learning set (ascending).

| Nr. | Quadrant | Model | Quality for test set | Error for test set |
|---|---|---|---|---|
| 1 | I-IV | RBF 19:19-15-4:4 | 0,9140 | 0,0818 |
| 2 | I | RBF 14:14-5-1:1 | 0,8923 | 0,1210 |
| 3 | II | RBF 12:12-5-1:1 | 0,8888 | 0,1434 |
| 4 | IV | RBF 10:10-10-1:1 | 0,7255 | 0,0776 |
| 5 | III | RBF 13:13-10-1:1 | 0,6890 | 0,0722 |

**Table S15.** Matrix of variables for the individual ANN models used.

| | | Ćwiartka | I | II | III | IV | I-IV | Number of occurrences of the variable |
|---|---|---|---|---|---|---|---|---|
| | | Model | RBF 14:14-5-1:1 | RBF 12:12-5-1:1 | RBF 13:13-10-1:1 | RBF 10:10-10-1:1 | RBF 19:19-15-4:4 | |
| | | Zmienne | | | | | | |
| Variables for all quadrants | | 1 | GENDER | GENDER | GENDER | GENDER | GENDER | 5 |
| | | 2 | AGE | AGE | AGE | AGE | AGE | 4 |
| | | 3 | AV | AV | AV | AV | AV | 5 |
| | | 4 | CQ | CQ | CQ | CQ | CQ | 3 |
| | | 5 | CS | CS | CS | CS | CS | 4 |
| | | 6 | CU | CU | CU | CU | CU | 2 |
| | | 7 | CW | CW | CW | CW | CW | 4 |
| | | 8 | CY | CY | CY | CY | CY | 2 |
| | | 9 | DA | DA | DA | DA | DA | 1 |
| | | 10 | DC | DC | DC | DC | DC | 4 |
| | | 11 | DE | DE | DE | DE | DE | 3 |
| 4CW | CW I | 12 | DG | - | - | - | DG | 1 |
| | | 13 | DW | - | - | - | DW | 2 |
| | | 14 | ED | - | - | - | ED | 2 |
| | | 15 | EL | - | - | - | EL | 1 |
| | CW II | 12 | - | DK | - | - | DK | 2 |
| | | 13 | - | DY | - | - | DY | 2 |
| | | 14 | - | EE | - | - | EE | 2 |
| | | 15 | - | EP | - | - | EP | 2 |
| | CW III | 12 | - | - | DS | - | DS | 2 |
| | | 13 | - | - | EC | - | EC | 2 |
| | | 14 | - | - | EG | - | EG | 2 |
| | | 15 | - | - | EX | - | EX | 2 |
| | CW IV | 12 | - | - | - | DO | DO | 2 |
| | | 13 | - | - | - | EA | EA | 2 |
| | | 14 | - | - | - | EF | EF | 2 |
| | | 15 | - | - | - | ET | ET | 2 |
| Output | | | DI | - | - | - | DI | |
| | | | - | DM | - | - | DM | |
| | | | - | - | DU | - | DU | |
| | | | - | - | - | DQ | DQ | |
